# Ecocentrism vs. Anthropocentrism: To the Core of the Dilemma to Overcome It

**DOI:** 10.1177/00243639251339844

**Published:** 2025-06-10

**Authors:** Patrícia Frantz, Francisca Rego, Stela Barbas

**Affiliations:** 1 Department of Bioethics, Faculty of Medicine, 26706 University of Porto, Porto, Portugal

**Keywords:** philosophy, environmentalism, theology, morality, ethics, speciesism

## Abstract

This article addresses the central moral challenge of twenty-first-century societies: identifying a core principle to guide the hierarchy of values in the context of environmental and human rights conflicts. It examines the ontological questions underlying these debates, such as the explanation of the world and the basis for action, emphasizing the human tendency to project values through either ecocentric or anthropocentric perspectives. This essay argues that resolving the tension between these two worldviews requires moving beyond their dichotomy by incorporating a third vertical axis inspired by the concept of *summum bonum* from classical philosophy. By revisiting this fundamental principle, this study proposes a framework for harmonizing humanity's relationship with its environment while respecting the dignity of all beings.

## Ecocentrism and Anthropocentrism

In moral and philosophical debates, different worldviews determine how we assign value to human and nonhuman entities. Anthropocentrism places humans at the center of moral concern, viewing the natural world primarily as a resource for human benefit. This paradigm has dominated Western thought since the modern era, projecting humanity as the primary agent of value and moral reasoning. By contrast, ecocentrism expands moral consideration to ecosystems as a whole, emphasizing the intrinsic worth of all living and nonliving components of nature. Closely related, biocentrism focuses on the value of all living beings, recognizing their right to exist independently of human needs. Meanwhile, theocentrism, which often underpins religious environmental ethics, places God or the divine as the ultimate source of value, with humans and nature positioned within a sacred hierarchy.

Even in this postmodern era, anthropocentric traits remain pervasive. The main object of moral concern continues to be man, as he directs the beam of attention he himself projects. However, this paradigm, which has prevailed throughout the modern era, has been increasingly challenged in intellectual circles. In the eighteenth century, the influential philosopher Jean-Jacques Rousseau (1712–1778) criticized civilization, praised the goodness of man in his natural state, and called for a return to nature (Rousseau 2012). Yet, above all, it was the effects of the Industrial Revolution that sparked the rise of environmental consciousness, culminating in the establishment of the first environmental organizations in the United States at the end of the nineteenth century.

In 1948, the year the Universal Declaration of Human Rights was signed, the first international environmental organization, the International Union for the Protection of Nature, was founded. The 1960s intensified this movement, merging it with countercultural currents symbolized by Woodstock. A pivotal moment came with Rachel Carson's *Silent Spring*, which underscored the ecological damage caused by pesticides like DDT (Carson 1962). In the 1970s, groundbreaking studies on acid rain by Likens and Bormann (1974) further expanded awareness of environmental degradation. Concurrently, global concerns about overpopulation, resource scarcity, and nuclear conflict were debated, notably by organizations such as the Club of Rome (Meadows, Randers and Meadows 2004).

Amid this so-called ecological crisis, the field of environmental ethics emerged, questioning the status of humanity and the value of nature. These debates laid the foundation for the theoretical pillars of environmental protection, bringing to the forefront a pressing question: What moral framework should guide our relationship with the natural world?

Environmental theorists have long argued that all living entities in nature possess intrinsic value—that is, they are ends in themselves and not merely instruments for achieving specific purposes. Recognizing such intrinsic value would, in turn, entail moral obligations for members of the human community. However, this raises a fundamental question: What criterion determines intrinsic value? For Immanuel Kant (1724–1804),^1^ rationality serves as the defining criterion for intrinsic value. In his framework, rational beings—humans—are ends in themselves, while all other entities lacking reason are reduced to the status of means, with merely instrumental value (Kant 2017). Kant's anthropocentric stance reflects the rationalist outlook of early modernity, which, as Dictoro highlights, regarded nature as an inert, mechanistic system, moved only by external forces (Dictoro et al. 2019).

In contrast, contemporary philosopher Peter Singer (1946–), following the utilitarian tradition of Jeremy Bentham (1748–1832), proposes sentience—the capacity to feel pleasure or pain—as the criterion for intrinsic value. Singer's approach significantly broadens the moral community, extending ethical consideration to nonhuman animals (Singer 2011). Moving further, Christopher Stone (1937–2021) argued that even trees and other natural objects deserve recognition as legal subjects, laying the foundation for modern environmental law (Stone 2010).

From the 1970s onward, environmental ethics diversified into various schools of thought. Among them, German philosopher Hans Jonas (1903–1993) advanced an anthropocentric approach grounded in responsibility. In his influential work *The Principle of Responsibility: Ethics for the Technological Age* (Jonas 1985), he articulated a moral imperative: “Act in such a way that the effects of your actions are compatible with the continuation of genuine human life on earth.” For Jonas, responsibility is an ontological value intrinsic to human existence. He argued that individuals have a duty toward the weakest, and it is through responsibility—rather than freedom—that people discern the boundaries of good and evil. Jonas's emphasis on precaution underscored humanity's obligation to future generations.

Similarly, political philosopher John Rawls (1921–2002) addressed the ethical implications of intergenerational justice in his “just savings principle” outlined in *A Theory of Justice* (Rawls 1999). Rawls asserted that societies must recognize the importance of preventing environmental damage that could undermine the health and opportunities not only of present but also of future generations. This principle further reinforced the ethical foundations for policies aimed at sustainability and fairness.

Faced with the environmental crisis and the looming scarcity of natural resources, prominent figures in the environmental movement, such as Arne Naess (1912–2009) and J. Baird Callicott (1941–), advocated for a drastic reduction in the global population (Varandas 2012). Similarly, Paul Ehrlich (1932–), in *The Population Bomb* (Ehrlich 1970), proposed controversial measures such as sterilization and the legalization of abortion to address overpopulation. However, this approach raises ethical concerns, as it seemingly undermines the value of human beings as inherently special.

Indeed, many environmentalists, drawing on the philosophy of Peter Singer (Singer 2015), argue that viewing humanity as unique constitutes *speciesism*—a form of species-based discrimination analogous to racism or sexism. From this perspective, humans are not inherently superior to other beings. Proponents of this view often align with the biocentric current, which advocates for ontological equality among all living beings. For biocentrists, the criterion for moral evaluation extends beyond rationality or sentience; it includes the simple fact of being alive.

Albert Schweitzer (1875–1965), a philosopher, theologian, and physician, developed an ethic of reverence for life. According to Brabazon, Schweitzer argued that all living beings deserve moral consideration and should be helped, not harmed (Brabazon 2000). This principle rejects hierarchical distinctions between species, promoting a universal respect for life. Similarly, Paul Taylor (1923–2015) advanced the notion of biocentric egalitarianism. For Taylor, life itself is the basis of intrinsic value, as each being exists to fulfill its own good. The *telos* of every organism's existence is its vitality—the drive to live—and this, he argued, grants all living beings an inherent dignity (Taylor 2011).

Biocentric authors challenge the notion of human superiority, questioning why rationality should be regarded as the ultimate moral criterion. Instead, they propose a paradigm shift: recognizing that the intrinsic value of life is not exclusive to humans but shared by all living entities. This perspective calls for a reevaluation of humanity's place within the broader web of life, rejecting anthropocentrism and advocating for a more inclusive ethical framework.

Another nonanthropocentric current of thought, in contrast to biocentric egalitarianism, advocates for *ecocentrism*. In this worldview, the planet as a whole—its ecosystem—becomes the central focus of moral considerations, representing the ultimate reference point for value. This perspective, initially proposed by environmental philosopher Aldo Leopold (1887–1948) and later expanded by Arne Naess within the Deep Ecology movement, views the planetary ecosystem as the moral community to which all beings belong. Humans are not privileged members but are instead one among many parts that have emerged through evolutionary processes, interconnected within the intricate web of life (Naess 1973).

From this standpoint, individual living beings are seen as transient, localized manifestations of the universal flow of energy. The symbiotic network of relationships that binds them constitutes their foundation and mode of existence. Ecocentrism rests on the ontological—that is, metaphysical—premise that there is no hierarchy of intrinsic value between human and nonhuman nature. Human existence is not exceptional but merely one component within the greater ecological tapestry. As ecocentrists argue, the Earth takes precedence because it predates humanity, which is a relatively recent arrival in the planet's long history.

The moral value of each element within the ecosystem, therefore, is measured by its contribution to the collective whole. The community takes precedence over individual components, as Farias and Torres point out: the parts derive their significance from their integration into the Whole (Farias and Torres 2014). Leopold envisioned this shift in perspective as an evolutionary step in ethics (Leopold 1949). In his view, humans must move beyond the role of conquerors and embrace their position as members of the biotic community. The ultimate good, according to ecocentrism, is the health and self-sustainability of ecosystems—a concept referred to as *autopoiesis*. As Maturana and Varela articulate: “Something is good if it preserves the balance, beauty, and integrity of the biotic community” (Maturana and Varela 1997).

This emphasis on preserving the balance and harmony of ecosystems provided the philosophical backdrop for practical environmental initiatives. For instance, the first United Nations Conference on the Environment, held in Stockholm in 1972, reflected the growing concern for aligning economic development with environmental preservation. This event marked a turning point in global awareness of the need to safeguard planetary integrity while fostering sustainable development (United Nations Environment Programme 1972).

**Figure 1.**
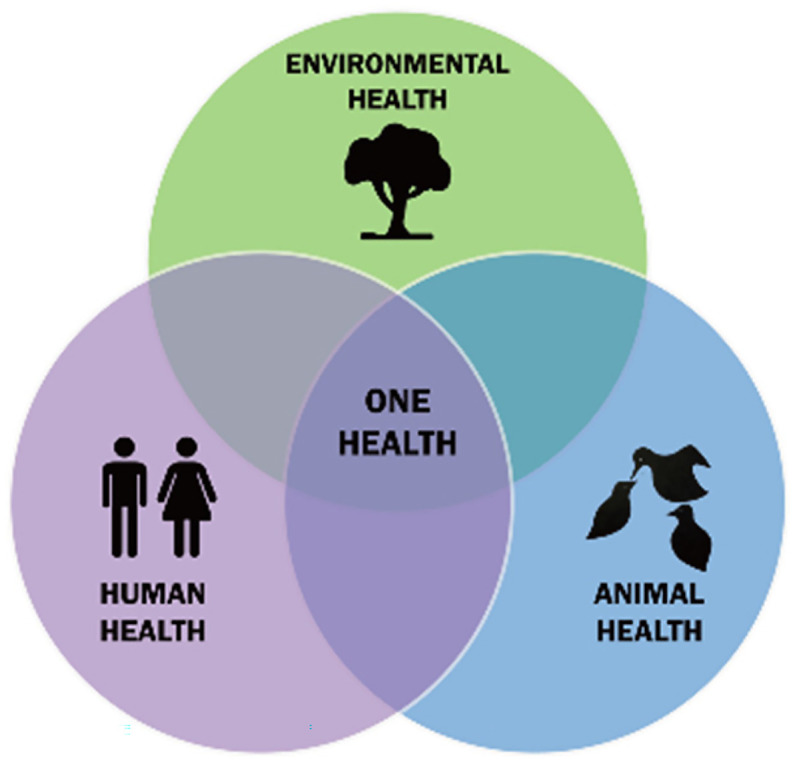
One Health triad. Wikimedia Commons.

However, some ecocentric activists have criticized the sustainability movement, labeling it as implicitly anthropocentric. Inspired by Arne Naess, movements such as Greenpeace and Earth First! have advocated for the ontological right of all beings to live and flourish (*self-realization*), promoting a simple, nature-based, pacifist, and anticonsumerist lifestyle (Varandas 2017). More recently, concerns over climate emergencies and biodiversity loss have intersected with growing awareness of human health risks, such as zoonoses. In response, the One Health Initiative Task Force proposed a new ethic in 2008, uniting human, animal, and environmental health under a single integrated framework (American Veterinary Medical Association 2008). This interdisciplinary approach seeks to bridge gaps between these three domains of care and highlights their interdependence, as represented in Figure 1.

The *One Health* framework attempts to harmonize anthropocentric, biocentric, and ecocentric perspectives, offering an integrated vision of human health alongside planetary and nonhuman well-being. However, this multidimensional approach raises critical questions: Can it truly help humans recognize the dignity of other life forms and the planetary ecosystem? Or does it risk perpetuating the dehumanization implicit in certain premises of biocentric and ecocentric ideologies? Is there a unifying point of reference—a transcendent good—that can guide humans in their relationships with one another, with other beings, and with the Earth?

These doubts arise precisely because of the multidimensional and relativistic nature of the postmodern worldview. Is it possible to articulate these dimensions cohesively? Around what central value will they converge? While the *One Health* framework suggests “health” as the intersection of these domains, the challenges of resolving conflicts between values persist. When all spheres—human, animal, and environmental—are placed on the same hierarchical level, goods and values risk becoming relativized. For instance, radical antispeciesist egalitarianism might lead to logically consistent yet intuitively absurd conclusions, such as equating the moral value of a bacterium to that of a human being.

Such dilemmas reflect deeper epistemological and philosophical tensions. The modern tendency to deconstruct hierarchies and subordination stems from centuries of intellectual movements, including Nominalism, Cartesianism, and Empiricism, which have profoundly shaped contemporary thought. This flattening of moral hierarchies, while aiming to promote equality, often obscures the specific and profound differences between humans and other living beings. Without a clear reference point to organize relationships and values, ethical frameworks may struggle to balance the inherent dignity of human life with the broader needs of the planet and its ecosystems.

## Three-Dimensional Polarization and Revisitation of Classical Philosophy

To overcome the intellectual and moral obstacles in addressing humanity's relationship with the environment, revisiting the reflections of classical philosophy can be illuminating. Aristotle, in *De Anima*, asserts that being animate represents a higher form of existence compared to inanimate beings (Aristotle 2017). For the ancients, the principle of motion inherent in living beings was attributed to the soul, conceived as the substantial form and the formula for their operation. Aristotle distinguished three levels of the soul based on their functions: (1) the *vegetative soul*, the most basic principle, governs processes such as generation, nourishment, and growth; (2) the *sensitive soul*, present in animals, enables the perception of the inner and outer worlds through sensory organs, granting a greater form of existence; and (3) the *intellectual soul*, unique to humans, endows them with the capacity for understanding, reflection, and self-awareness.

This hierarchy underscores the exceptional nature of human participation in being, a difference that cannot be ignored in moral considerations. Humans hold a distinct position in the hierarchy of living beings as the only species capable of reflecting on its place in the cosmos. This observation serves as a critical counterpoint to ecocentric worldviews that minimize human significance.

From this perspective, one can discern a latent misanthropy and pessimism inherent in certain ecocentric doctrines, as noted by Callicott (1999). If humanity is perceived merely as one part of the Earth's ecosystem—devoid of intrinsic meaning—it risks being reduced to an incidental or even detrimental element. This perspective is exacerbated by some proponents of ecocentrism who advocate for drastic population reduction through controversial means such as sterilization and abortion (Naess 1973). Such views echo earlier ideas from figures like Ernst Haeckel (1834–1919), a prominent biologist and ecologist who controversially justified eugenics and scientific racism under the guise of social Darwinism (Weikart 2004).

The neo-Malthusian population control movement of the twentieth century perpetuated this line of thinking. Influential voices such as Ehrlich, in his book *The Population Bomb*, compared population growth to a cancer that threatens the planet (Ehrlich 1970). Figures such as Bertrand Russell, Isaac Asimov, Jacques Cousteau, and Margaret Sanger expressed similar concerns, with some explicitly advocating eugenics as a means of controlling population numbers (Eliot and Eliot 1921). Population control policies, often targeting the so-called Third World, were supported by powerful governments (Kissinger 1974) and international foundations such as Rockefeller and Ford, ostensibly to alleviate poverty (Derosa 2018). However, these efforts were often rooted in a nihilistic worldview that equated human existence with environmental and economic degradation.

Despite the alarmist predictions of neo-Malthusian theorists, reality has proven many of their assumptions wrong. Food production has grown exponentially, challenging the core premise of Malthusian theory (Richie, Rosado and Roser 2022). Moreover, contemporary concerns about demographic decline, reflected in historically low fertility rates in many countries (GBD 2021 Fertility and Forecasting Collaborators 2024), suggest that the problem today is not overpopulation but its opposite. Ultimately, the pessimism and reductionism of these worldviews fail to account for the human capacity for innovation, reflection, and moral agency. While it is undeniable that humanity must act responsibly toward the planet, any ethical framework must also recognize the unique dignity and creative potential of human beings.

## *Homo Faber* and the Paradox of Human Intervention

Humans, as *homo faber*, are distinguished by their capacity for poietic reason—the ability to create and transform the natural environment. Yet, this very capacity often leads to actions that harm not only nature but also humanity itself. Since the Industrial Revolution, we have witnessed the unprecedented consequences of greed and selfishness in shaping the relationship between individuals, society, and the environment. Technology, while indispensable for human life, inherently carries dual effects: it brings benefits and harms that must be carefully evaluated by society. This raises the question: what is the most ethical and effective way for humanity to address the challenges of its own creations?

The Brazilian writer Gustavo Corção insightfully described humans as “artificial beings by nature” (Corção 2019). Indeed, despite aspirations for a harmonious existence with nature, virtually all aspects of modern life depend on human intervention and ingenuity: from food production and housing to clothing, medicine, and beyond. This reality fuels the critique of ecocentrists, who argue that humanity often behaves as if it were separate from nature. They highlight the conquering and exploitative tendencies of human behavior. However, it does not logically follow that fewer humans would solve the problem. While some individuals misuse technology to harm the environment, others use it to innovate and improve living conditions. Technological progress has undeniably elevated global living standards.

Paradoxically, the environmental crisis, though sometimes marred by alarmism and radicalism, has spurred significant advances in conservation and sustainability. However, tensions persist between the interests of individuals and communities and those who position themselves as guardians of environmental rights. These conflicts highlight the complexity of balancing human well-being with environmental preservation.

## Environmental Rights and the Question of Foundations

Environmental law is still evolving, largely due to unresolved philosophical debates about the nature of rights. What does it mean to be a subject of rights? Who can hold these rights? Does the environment itself have rights, or is this concept limited to sentient beings? Legal positivism asserts that law is a human construct, deriving its value from its creator: humanity. As Comparato argues, the foundation of rights is none other than the dignity of the human person, which precedes and supersedes any group-specific or individual specifications (Comparato 1998).

Even biocentric thinkers like Taylor concede that plants and animals lack rights in the strict sense because they cannot exercise them (Taylor 2011). In the absence of a more robust philosophical foundation, many legal frameworks have focused on protecting public health, a goal that inadvertently reinforces the anthropocentric paradigm critiqued by ecocentric environmentalists. Moral attributions such as sentience (the capacity for pleasure or pain) and intrinsic value are, by necessity, filtered through human perception. For example, the equal consideration of the well-being of all entities—human and nonhuman—or the recognition of intrinsic value in ecosystems ultimately depends on human judgment.

In light of these ambiguities, some thinkers have called for a reevaluation of the philosophy of nature. Lovelock famously proposed the Gaia hypothesis,^2^ which posits that Earth functions as a living system (Lovelock 1979). However, such claims require careful philosophical scrutiny and clarification. Regardless of the framework adopted, the evaluation of rights, values, and moral principles remains rooted in humanity's unique capacity for rationality. Thus, the primary concern should be understanding the hierarchies of values that humans establish and ensuring that these hierarchies align with both the dignity of human life and the ethical care for the environment.

## The Necessity of Reflection on the Good

Given humanity's profound capacity to intervene in and transform the environment, the need for reflection on the concept of the good becomes paramount in the search for harmony. As Aristotle articulated in the *Nicomachean Ethics*, human actions are oriented toward multiple ends, and these ends must be subordinated to one another in a coherent hierarchy (Aristotle 1996). However, modern society struggles to recognize the interconnection and subordination of these ends.

In our contemporary worldview, there is a tendency to adopt a horizontal perspective. On one hand, this perspective aligns with the idea of a “deep cosmic unity,” ^3^ as proposed by deep ecology, where all entities are interconnected. On the other hand, it can manifest as a fragmented, Cartesian^4^ view, where each entity is analyzed separately and in isolation. This division resembles the way particles are studied at the subatomic level: some focus on individual particles, while others study their compounds. Both approaches reflect different forms of materialism.

**Figure 2.**
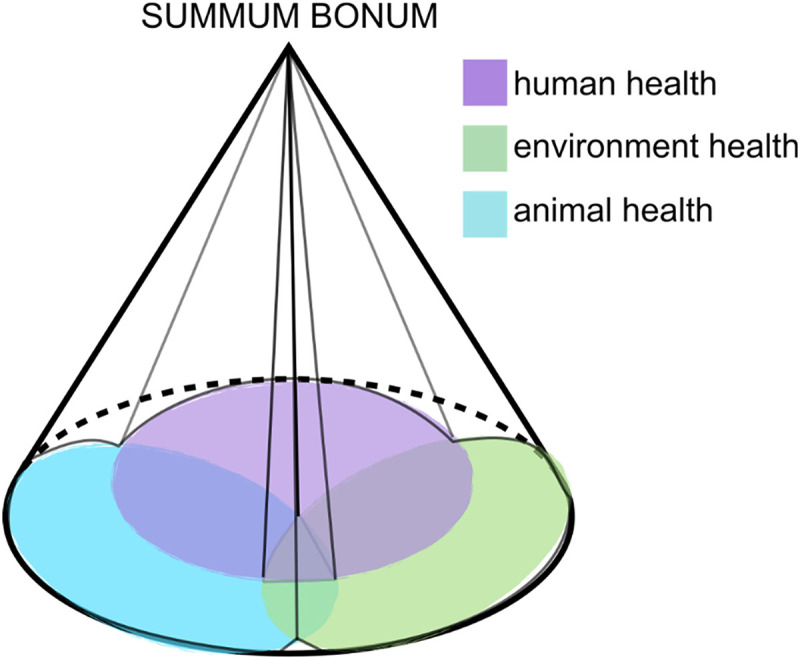
Three-dimensional polarization.

The crucial question, however, is what brings these particles together? What shapes matter and gives each thing its distinct identity? A purely horizontal perspective fails to capture the entirety of reality, as it obscures the unity and purpose that underlie the world. By adopting a vertical perspective, we might better grasp the hierarchical order of goods and the relationships between them. Such an approach points to something above, a higher principle that governs the subordination and unity of all things. This “above” cannot be reduced to the towering skyscrapers of modern mega-metropolises (Figure 2). Instead, it represents a transcendent foundation that provides meaning and coherence to the relationships between beings and the goods they pursue.

## The Third Way: From Dualism to Theocentrism

After the brief remark on the hierarchy of goods, it is worth considering a third way to overcome the impasse between ecocentrism and anthropocentrism. Human reason measures goods, but man is not the supreme good to which all things are subordinated. Is man capable of achieving the highest good? As Reale points out, for Plato, the supreme good would be unattainable, belonging to the world of the Forms; for Aristotle, the supreme good, identified with happiness, could be sought and found by men (Reale 1990). However, Aristotle's metaphysics incorporates Plato's idea that all movement refers to being in pure act, the First Unmoved Mover (God). Later, Augustine synthesized the Platonic Good with Christianity, formulating the idea of God as the *summum bonum*^5^ (Moon 1955).

This third way, theocentrism, proposes the recognition of a supreme good that orders and directs all things. Instead of a horizontal and chaotic perspective, where the notion of priority is lost, theocentrism offers a vertical outlook, with goods subordinated to the highest good, enabling us to clearly discern excellent actions that maintain the harmony (*kosmos*) of the whole. Humanity has already had enough time to experience the rise of anthropocentrism, which has paradoxically led to the devaluation of man and reason. Therefore, the proposal is not to return to feudalism or the Middle Ages, but perhaps to recover something from the ancient understanding of cosmic order that was lost in modernity.

Authors such as Hoffman and Sandelands suggested this in *Getting Right with Nature: Anthropocentrism, Ecocentrism, and Theocentrism*, pointing out how the moral confusion between man and nature reflects an inherently unstable metaphysics (Hoffman and Sandelands 2005). In anthropocentrism, the human subject takes precedence over the object (nature); in ecocentrism, nature as the substrate takes precedence—this naturalism ultimately leads to subjectivism, where what matters is only what the individual thinks or values. This subjectivism, exacerbated in postmodernism, results in the loss of essence and the idea that reality is socially constructed. As Martin Krieger notes, even the value of something like Niagara Falls would be “socially constructed” (Varandas 2012).

This blindness has roots in the nominalism of William of Ockham,^6^ who denied the existence of universals, reducing them to mere names (Ockham 2011). Nominalism influenced empiricism, which in turn fragmented reality into its sensible and measurable aspects, enhancing human control over matter but obscuring the transcendent value of things. However, the theocentric metaphysics offers an integrated view: man and nature are seen in relation to God. As St. Thomas Aquinas stated, “God speaks in words and things.” In this sense, writer G.K. Chesterton recalls St. Francis of Assisi, who saw nature as a sister, because we are all children of the same Father. In *The Canticle of the Creatures*, the saint praised even the smallest of God's creations as brothers. Therefore, neither anthropocentrism nor ecocentrism truly reconciles man with nature. Only theocentrism, by ordering both man and the world in relation to the supreme good, can offer a harmonious and integrated vision of reality.

By perceiving “the hands of God” in creation, we recognize that our relationship with nature is not merely a subject-object dynamic but a union in God. As John Paul II emphasizes in his encyclical *Evangelium Vitae*, the meaning of things becomes distorted when any reference to God is removed (Pope John Paul II 1995). Nature is reduced to mere matter, subjected to every form of manipulation, including that of man. Similarly, the opposite stance, which denies any intervention in nature and attributes everything to God's will, also falls short. In this “green spirituality,” Gaia—Mother Earth—is worshipped as if she were a deity.

Only a metaphysics that transcends the eco-anthropocentric dualism can offer a deeper understanding of the value of natural resources and environmental ethics. It is through such a framework that we can grasp the virtues necessary for humanity's relationship with the natural world. Hoffman and Sandelands (2005), for instance, highlight the importance of humility. Without recognizing the limits of human action and knowledge, our interference remains minimal, often driven by an inadequate understanding of cause and effect, with unpredictable and sometimes irreversible consequences for humans and other living beings. As Paul VI noted, “The most extraordinary scientific progress, the most astonishing technical inventions, the most tremendous economic development, if they are not linked to social and moral progress, inevitably turn against man” (Paul VI 1968).

## Final Considerations

Humanity was created to tend to the “garden.” We are the beings entrusted with the care of this “common home,” as Pope Francis calls it in his encyclical *Laudato Si’* (Francis 2015). Nature serves us, yet we also have a responsibility to serve it. Whatever mankind creates through its artistic capacity, it is always dependent on what has been given to us. Man does not create himself, nor does he create *ex nihilo*. He exists in a real, physical world that provides everything he needs to live. He is a tribute to this nature, as are his family, other people, his culture, and civilization. As Francis says, “The waste of creation begins where we no longer recognize any authority over ourselves but see only ourselves.”

From a theocentric perspective, it seems wrong to deny other beings in nature an intrinsic value and to view them merely as objects of utility. Like humans, they simply *are.* Interestingly, the value humans assign to them is also important. If man is attentive, he can perceive beauty and feel the sublime in nature, understanding that this wonder is not confined to himself but points to something beyond. By contemplating his essence and the world, man recognizes the profound mystery of life and existence. He realizes that, despite his spectacular inventions, he cannot create life. None of this could exist on its own, as the beings of the world are not necessarily like mathematical formulas; they are contingent. Man, a being endowed with reason, could not exist in isolation—and it is tragic to imagine a world without a being capable of admiring and caring for such beauty. Who, if not man, can truly contemplate and appreciate nature?

However, above man stands the *Logos*^7^—the principle of order that man seeks to understand. To resolve the polarization between ecocentrism and anthropocentrism and its inherent contradictions, it seems necessary to include a third, fundamental vertical point above the two opposing perspectives. The theocentric worldview, though often excluded from public environmental discourse, is the only one capable of harmoniously reconciling humanity with nature.

## Notes

1. Immanuel Kant (1724–1804), in his *Groundwork of the Metaphysics of Morals*, argues that rational beings possess an intrinsic worth or dignity (*Würde*), which arises from their capacity for moral reasoning and autonomy. This dignity means they should never be treated solely as tools for others' purposes.

2. The Gaia Hypothesis, developed by James Lovelock in the 1970s, posits that Earth and its biological systems act as a self-regulating entity. In some environmental philosophies, this concept has been interpreted metaphorically or spiritually, leading to the idea of “Gaia” as a living goddess.

3. Deep ecology is an environmental philosophy that promotes the intrinsic value of all living beings, regardless of their utility to humans. It was developed by the Norwegian philosopher Arne Naess in the 1970s.

4. Cartesian dualism, proposed by René Descartes (1596–1650), is the philosophical view that reality is composed of two fundamentally distinct substances: mind (*res cogitans*), which thinks, and matter (*res extensa*), which occupies space.

5. *Summum bonum* is a Latin phrase meaning “the highest good.” In classical philosophy, particularly in Aristotle and later Christian thought (e.g., Augustine), it refers to the ultimate purpose or final good that gives meaning and direction to human life.

6. Nominalism, a doctrine associated with William of Ockham (1287–1347), rejects the existence of universal forms or essences (e.g., "humanity") as independent realities. Instead, universals are seen as mere names (*nomina*) that the human mind uses to group individual objects.

7. *Logos* is a Greek term meaning “word,” “reason,” or “principle.” In ancient Greek philosophy (e.g., Heraclitus), it refers to the rational principle that governs the cosmos. In Christian theology, *Logos* is used to describe Christ as the divine Word through which all things were created (John 1:1).
